# In Vitro Activity of Newer and Conventional Antimicrobial Agents, Including Fosfomycin and Colistin, against Selected Gram-Negative Bacilli in Kuwait

**DOI:** 10.3390/pathogens7030075

**Published:** 2018-09-17

**Authors:** Wadha Alfouzan, Rita Dhar, David P. Nicolau

**Affiliations:** 1 Microbiology Unit, Department of Laboratories, Farwaniya Hospital, Farwaniya 81004, Kuwait; riitampdhar@gmail.com; 2 Department of Microbiology, Faculty of Medicine, Kuwait University, Kuwait City 13110, Kuwait; 3 Center of Anti-Infective Research, Hartford Hospital, Hartford, CT 06102, USA; david.nicolau@hhchealth.org

**Keywords:** in vitro activity, Gram-negative bacilli, antimicrobial agents, ceftolozane/tazobactam, ceftazidime/avibactam, fosfomycin, colistin

## Abstract

Limited data are available on susceptibilities of these organisms to some of the recently made accessible antimicrobial agents. The in vitro activities of newer antibiotics, such as, ceftolozane/tazobactam (C/T) and ceftazidime/avibactam (CZA) along with some “older” antibiotics, for example fosfomycin (FOS) and colistin (CL) were determined against selected strains (resistant to ≥3 antimicrobial agents) of *Escherichia coli*, *Klebsiella pneumoniae*, and *Pseudomonas aeruginosa*. Minimum inhibitory concentrations (MIC) were determined by Clinical and Laboratory Standards Institute microbroth dilution. 133 isolates: 46 *E. coli*, 39 *K. pneumoniae*, and 48 *P. aeruginosa* were tested. Results showed that *E. coli* isolates with MIC_50/90_, 0.5/1 μg/mL for CL; 4/32 μg/mL for FOS; 0.25/32 μg/mL for C/T; 0.25/8 μg/mL for CZA, exhibited susceptibility rates of 95.7%, 97.8%, 76.1%, and 89.1%, respectively. On the other hand, *K. pneumoniae* strains with MIC_50/90_, 0.5/1 μg/mL for CL; 256/512 μg/mL for FOS; 2/128 μg/mL for C/T; 0.5/128 μg/mL for CZA showed susceptibility rates of 92.3%, 7.7%, 51.3%, and 64.1%, respectively. *P. aeruginosa* isolates with MIC_50/90_, 1/1 μg/mL for CL; 128/128 μg/mL for C/T; 32/64 μg/mL for CZA presented susceptibility rates of 97.9%, 33.3%, and 39.6%, respectively. Higher MICs were demonstrated against most of the antibiotics. However, CL retained efficacy at low MICs against most of the isolates tested.

## 1. Introduction

It all began with an increase in the prevalence of *Enterobacteriaceae* producing extended-spectrum beta-lactamases (ESBLs) in the 1980s and 1990s. Earlier to that almost all *Enterobacteriaceae* were susceptible to broad-spectrum antibiotics, including beta-lactam/beta-lactamase inhibitor combinations, oxyimino-cephalosporins (e.g., cefotaxime, ceftriaxone, and ceftazidime), aztreonam, and carbapenems [1]. The evolution of ESBLs three decades ago significantly limited the efficacy of oxyimino-cephalosporins and aztreonam. Infections due to ESBL-producing *Enterobacteriaceae* (ESBL-PE) reached unprecedented levels in Europe [2,3,4,5] as well as in Asia [6]. These Gram-negative bacilli (GNB) are increasingly resistant to several antibiotics particularly broad-spectrum cephalosporins, because of global spread of ESBL-PE as well as AmpC cephalosporinase producing Enterobacteriaceae and *P. aeruginosa* [2,3,4]. Since carbapenems are currently considered the only beta-lactams to have activity against such isolates, this led to concomitant increased use or overuse of carbapenems between 2010 and 2014 as published in a recent 2016 ESAC report [7,8,9,10]. Thus, selective pressure for carbapenem resistance has spread progressively after emerging rapidly during the 1990s and continues to increase steadily worldwide, not only in nonfermenter GNB but also in Enterobacteriaceae [11].

The various mechanisms that are involved in the development of carbapenem resistance among GNB include (i) selective loss of external membrane permeability such as OprD porin loss in *P. aeruginosa*; (ii) the combination of impermeability with various broad-spectrum beta-lactamases (ESBL and or cephalosporinase); and (iii) carbapenem-hydrolyzing enzymes such as carbapenemases (e.g., *bla*_KPC_, *bla*_VIM_, *bla*_OXA-23_, *bla*_OXA-48_, *bla*_IMP_, and *bla*_NDM_). Since genes coding carpanenemases are carried by plasmids there is high potential for dissemination. Furthermore, carbapenem-resistant GNB are invariably also resistant to multiple drug classes, such as aminoglycosides, fluoroquinolones, and folate inhibitors, due to additional types of resistant genes carried by the organisms, leaving very few therapeutic options [12,13].

In order to reduce the risk of development of resistance to carbapenems two main strategies are considered: (i) investigating alternative treatments for ESBL-PE-related infections and (ii) antimicrobial de-escalation. The main aim remains to adopt a “carbapenem-sparing strategy” so that this group of antibiotics retains activity against ESBL-PE, especially those causing life-threatening infections. Several studies from Kuwait have highlighted the growing concerns over the rising incidence of ESBL-producing and carbapenem-resistant *Enterobacteriaceae* (CRE) in the region [14,15,16]. Since the treatment options for treating CRE are limited, focus is on novel agents to overcome the menace of multidrug resistant (MDR) GNB. Recently, agents from additional drug classes have demonstrated in vitro activity against ESBL-PE/CRE and these include β-lactam/β-lactamase inhibitor combinations, e.g., ceftolozane/tazobactam (C/T) and ceftazidime/Avibactam (CZA) [17].

This study was undertaken to investigate the antimicrobial susceptibility patterns of selected strains (those with MDR profile) of *E. coli*, *K. pneumoniae*, and *P. aeruginosa*, which were isolated from various clinical specimens. The antimicrobial agents tested included C/T and CZA in addition to other conventional and re-emerging older antibiotics including colistin (CL) and fosfomycin (FOS), which had fallen out of repute due to issues of efficacy, pharmacokinetics, and/or toxicity. Furthermore, previously reported data is compared and analyzed with the results obtained in the present study.

## 2. Materials and Methods

(1) *Bacterial isolates*: Nonduplicate (single isolate per patient) isolates of *E. coli* (n = 46), *K. pneumoniae* (n = 39), and *P. aeruginosa* (n = 48) from blood, respiratory, or urine samples, which showed resistance to ≥3 classes of antibiotics by disk diffusion or commercial methods, namely Vitek2 Compact System (BioMérieux Inc., Marcy-l’Etoile, France) or Phoenix (Becton, Dickinson & Co., Sparks, MD, USA) were selected for testing. Bacterial identification was based on the previously mentioned commercial systems, and confirmed when necessary by matrix-assisted laser desorption ionization-time of flight mass spectrometry (MALDI-TOF MS) Vitek MS (BioMérieux Inc., Marcy-l’Etoile, France) following the manufacturer’s instructions in microbiology laboratory at Farwania Hospital, Kuwait. The selected isolates were preserved in Tripticase soy broth with 20% glycerol at −80 °C [18].

(2) *Antimicrobial susceptibility testing*: All study isolates were shipped to Center for Anti-infective Research and Development (CAIRD) where antimicrobial susceptibility testing was performed by using broth microdilution panels, according to Clinical and Laboratory Standards Institute (CLSI) standards [18,19]. Thirteen antimicrobial agents were used for susceptibility tests in the present study, namely ceftazidime (CAZ), cefepime (FEP), imipenem (IPM), meropenem (MEM), piperacillin-tazobactam (PTZ), ciprofloxacin (CIP), aztreonam (AZM), tobramycin (TOB), and amikacin (AN) in addition to CL, FOS, C/T, and CZA. For all antimicrobial agents tested, susceptibility was based on CLSI clinical breakpoints except CL for which MIC was interpreted as Epidemiological Cutoff Value (ECV) and expressed as Wild Type (susceptible; MIC ≤ 2 μg/mL) or Not Wild Type (resistant; MIC ≥ 4 μg/mL) [20]. While FOS broth dilution minimum inhibitory concentrations (MICs) are not recommended by CLSI, studies have shown them to be similar to the agar dilution methods [21]. FOS broth MICs were determined after addition of glucose-6 phosphate to the nutrient medium, which potentiates the action of FOS against *E. coli* and *K. pneumoniae*, although such potentiation is not detectable with *P. aeruginosa* [22]. For CZA, avibactam was tested at a fixed concentration of 4 μg/mL in combination with doubling dilutions of CAZ and MICs were interpreted using U.S. FDA MIC breakpoints for Enterobacteriaceae, with susceptibility being read at MIC of ≤8 μg/mL and resistance at MIC of ≥16 μg/mL [23].

## 3. Results

The overall susceptibility and MIC_50_/MIC_90_ results of all the isolates tested are presented in Table 1 and Table 2, respectively. Among *E. coli* strains, only one of them was found to be uniformly susceptible to all the antimicrobial agents tested. However, there were two strains, which showed resistance to COL but remained susceptible to AN, FOS, IMP, MER, and PTZ. Five of the *E. coli* strains, which exhibited low MICs (≤0.125 μg/mL) against AZM showed similar results with FEP (MIC ≤ 0.25 μg/mL). Although 6.5% (3/46) of the isolates were resistant to AN, two of them presented with high MICs (≥256 μg/mL) and remained susceptible only to CL and FOS among the antimicrobials tested. 

Of 39 *K. pneumoniae* isolates only one was found to be susceptible to all the antimicrobials tested. Two of the four strains which appeared resistant to CL (MIC ≥8 μg/mL), showed susceptibility to carbapenems (one with intermediate susceptibility to IMP). Only two isolates showed activity against AZM (MIC ≤ 0.25 μg/mL) and FEP (MIC ≤ 0.5 μg/mL). In contrast to *E. coli*, 14/39 *K. pneumoniae* strains exhibited high MICS against AN (MIC ≥32 μg/mL) while 36/39 (92.3%) isolates were resistant to FOS (MIC >64 μg/mL). Also, 10 isolates with AN MICs of ≥512 μg/mL were resistant to all other antibiotics except CL with one strain showing resistance to CL as well.

**Figure 1:**
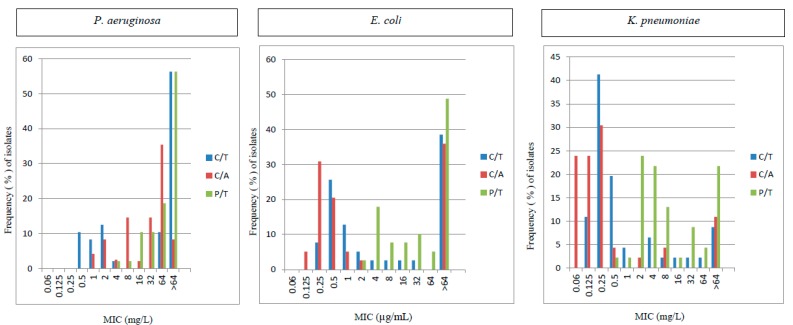
MIC of Ceftolozane/Tazobactam (C/T), Ceftazidime/Avibactam (C/A), and Piperacillin /Tazobactam (P/T) against *E. coli*, *K. pneumoniae* and *P. aeruginosa*.

Among *P. aeruginosa* isolates only one was found to be panresistant, including CL while 47/48 (98%) were at least susceptible to CL. Interestingly, AZM, FEP, and CL showed better activity (47.9%, 25.0%, and 97.9%, respectively) against *P. aeruginosa* than *E. coli* and *K. pneumoniae* isolates (Table 1). However, C/T appeared less potent against *P. aeruginosa* (33.3% susceptible) as compared to *E. coli* (76.1% susceptible) and *K. pneumoniae* strains (51.3% susceptible). Also, CZA showed a susceptibility rate of 39.6% against *P. aeruginosa* whereas 89.1% of *E. coli* and 64.1% of *K. pneumoniae* strains were susceptible (Table 1). Comparative MIC ranges for C/T, CZA and PTZ against *E. coli*, *K. pneumoniae*, and *P. aeruginosa* isolates are illustrated in Figure 1.

## 4. Discussion

Many studies in the medical literature illustrate the global increase in the burden of antibiotic-resistant Gram-negative pathogens. However, wide regional differences exist necessitating the need to take into account the local epidemiology of infections caused by MDR GNB as well as the antibiogram at the level of the country, the region, the hospital, and at times the individual hospital unit. However, the limited therapeutic options due to shortage of new antibiotics have increased the interest in “old antibiotics” such as FOS and CL [24]. Earlier in vitro studies have shown that FOS remains active against ESBL- and carbapenemase-producing *E. coli* and *K. pneumoniae* isolates and the drug has been approved by the US Food and Drug Administration for use in the treatment of uncomplicated urinary tract infections [25,26]. The mode of action of FOS is to inhibit bacterial cell wall biogenesis by inactivating the enzyme *Mur A* (UDP-Nacetylglucosamine-3-enolpyruvyl transferase), an enzyme essential for peptidoglycan synthesis. Excellent in vitro activity of FOS was reported against 68 KPC-producing *K. pneumoniae* strains with susceptibility rates of 93% for the overall group and 83% for the CL-resistant subgroup [27]. Similar to this finding, a more recent study on urinary isolates of *E. coli* and *K. pneumoniae* reported rates of susceptibility of *E. coli* to CL and FOS as 100% and 98.1%, respectively, and 100% and 95.5% for *K. pneumoniae* isolates, respectively. The MIC_50_ and MIC_90_ of FOS for *Enterobacteriaceae* (including *E. coli* and *K. pneumoniae*) were found to be 2 μg/mL and 8 μg/mL, respectively. Although the overall susceptibility of Enterobacteriaceae to FOS was 95.2%, the susceptibility rates of 95.9% and 89.1% were observed for MDR Enterobacteriaceae and CRE, respectively (MIC range, 0.25–512 μg/mL) [28]. However, these results are not in agreement with our observation, especially in regard to *K. pneumoniae* strains, which could be explained by use of different method (E test) for determining the MICs of FOS for Enterobacteriaceae other than *E. coli* and interpreting the results according to EUCAST guidelines 2015 as given by Falagas et al. [25] and perhaps the local epidemiology. Also, the MIC values were higher for both *E. coli* and *K. pneumoniae* isolates in our study (Table 2). However, in a recent study, which performed and interpreted MICs according to the CLSI, tested 16 antibiotics against 613 Enterobacteriaceae isolates (constituted of 56.9% *E. coli* and 17.8% *K. pneumoniae*) and demonstrated high susceptibility rates (92.8%) with FOS [29].

Although CL retained activity against ESBL-PE and CRE in initial studies [28], more recent data suggest that resistance to CL is emerging with reports even describing outbreaks caused by CL-resistant strains [30]. In our study CL performed better against *P. aeruginosa* isolates with a susceptibility rate of 97.9% than with *E. coli* (97.8%) and *K. pneumoniae* (92.3%) whereas Ip et al. showed a susceptibility rate of 88.8% against Enterobacteriaceae strains [28]. A study from Taiwan demonstrated that CL had only moderate in vitro activity (73% susceptible) against isolates of *P. aeruginosa* [31]. Another study from Turkey, which measured CL susceptibility against MDR GNB by E test (not recommended for by CLSI) showed that 51 *P. aeruginosa* isolates exhibited low MICs (range 0.25 to 2.0 μg/mL) [32]. In a study from Saudi Arabia susceptibility of 33 isolates of MDR *P. aeruginosa* was determined against CL, carbapenems, and tigecycline by E test following the CLSI breakpoint recommendation. However, their results showed that CL had excellent activity against 93.9% of the strains, a finding which can be corroborated by our data, probably because Saudi Arabia and Kuwait share the same geographic region and hence the same epidemiology [33,34].

In a large study from the US, that evaluated in vitro activity of C/T and comparator agents against Enterobacteriaceae and *P. aeruginosa* isolates from hospitalized patients, the most active agents against Enterobacteriaceae were found to be C/T, AN, and MER with susceptibility rates of 94.4%, 99.0%, and 98.0%, respectively. Although C/T demonstrated good activity against ESBL non-CRE phenotype strains of Enterobacteriaceae (87.5% susceptible), it showed poor activity against CRE strains. For *P. aeruginosa* isolates effectiveness of C/T and CL was comparable, with susceptibility rates of 97.3% and 99.5%, respectively, with C/T maintaining activity against MDR-*P. aeruginosa* (88.6% susceptible) as well [35]. In a similar study from the Asia-Pacific, using CLSI breakpoints, it was revealed that C/T and MEM were the most potent compounds tested against Enterobacteriaceae isolates, with susceptibility rates of 89.2% and 96.3%, respectively. Whereas C/T showed good activity against ESBL non-CRE phenotype of Enterobacteriacae (MIC_50/90_, 0.5/16 μg/mL) it did not prove effective against isolates with CRE phenotype (MIC_50/90_, >32/>32 μg/mL) [36]. On the other hand, comparable susceptibility rates of 90.8%, 91.2%, and 98.4% were obtained with C/T, AN, and CL, respectively against *P. aeruginosa* isolates (35). In comparison, our strains of *P. aeruginosa* with MIC_50/90_ and 128/128 μg/mL for C/T exhibited susceptibility rates of 33.3% and 97.9% with C/T and CL, respectively. Also, 15 (31.2%) of *P. aeruginosa* strains, which were resistant to IMP/MEM tested susceptible to C/T, whereas only 8.3% of the strains exhibited resistance to C/T while retaining susceptibility to IMP/MEM.

In an analysis of in vitro activity data on CZA tested against Enterobacteriaceae and Pseudomonas isolates, it was shown that this agent was very active against all of Enterobacteriaceae isolates, with overall MIC_50_ and MIC_90_ values of 0.25 and 1.0 μg/mL, respectively [37]. However, *K. pneumoniae* isolates, which were class B metallo-β-lactamase (not inhibited by avibactam) producers were inhibited at a higher concentration (MIC ≥32 μg/mL) of CZA. The majority of the isolates, which either possessed ESBL enzymes (CTX-M, OXA-1/30 or SHV-12) or carbapenemases (KPC, OXA-48), were inhibited by CZA at MIC ≤8 μg/mL. Among the Pseudomonas isolates tested, MIC_50_ and MIC_90_ values for CZA were reported as 8 and 64 μg/mL, respectively. Higher MICs were attributed to the presence of Class B or Class D enzymes [36,37]. These findings are in contrast to an earlier study, which showed better anti-pseudomonal activity of CZA (93.7% susceptible) compared to IMP (85.7%) or PTZ (74.6%) [38]. Our data showed that the susceptibility rates of 89.1% among *E. coli* strains against CZA (MIC_50/90_, 0.25/8 μg/mL) were less than that observed with AN (91.3%), CL (95.7%), and FOS (97.8%). In comparison CZA appeared less potent against *K. pneumoniae* (64.1% susceptible) and *P. aeruginosa* (39.6%) isolates.

In another similar study, 96.9% of Pseudomonas isolates were reported as susceptible to CZA (MIC ≤8 μg/mL) while the susceptibility rates for CAZ, MEM, and PTZ were 83.8%, 81.9%, and 78.5%, respectively [39]. The discrepancy between our results and other studies could be explained by the fact that most of our isolates were of MDR profile, whereas the majority of other studies tested unselected strains. However, our findings were comparable to that of Stone et al. who showed that the CAZ and CZA MIC_50/90_ values for 27 *P. aeruginosa* isolates were 64/> 64 μg/mL and 8/64 μg/mL, respectively, versus 64/128 μg/mL and 32/64 μg/mL in the present study. Higher MIC_90_ values were assessed to be due to the possession of either a class B or a class D enzyme as those strains which lacked these enzymes had a MIC_90_ value of 8 μg/mL [40].

## 5. Conclusions

Antimicrobial resistance is a persistent global threat and continuous monitoring of evolutionary trends in the susceptibility patterns of GNB causing wide spectrum of infectious diseases is mandatory. The present study demonstrates that older antimicrobial agents, such as CL and FOS, are still potent against selected GNB isolated from our hospital. CL is the major representative of revived antibiotics with the broadest spectrum of activity against MDR GNB although a wide range of efficacy has been reported ranging between 25% and 71%. On the other hand, FOS was found to be effective only against MDR *E. coli* strains in our study unlike earlier studies claiming it to be a treatment option for infections caused by both *E. coli* and *K. pneumoniae*. Further investigation by studies evaluating the in vitro activity of these agents must be confirmed before clinical use. While emerging drugs such as C/T and CZA may also be useful by offering additional therapeutic alternatives with fewer side effects and toxicities than agents such as the polymyxins, important issues still remain. First, additional investigation is needed to firmly establish the efficacy of monotherapy versus combination therapy and the efficacy of specific agents. Second, more study is needed to determine the appropriate dosing regimen for these agents, from both clinical efficacy and toxicity perspectives. Third, the best antimicrobial stewardship practices to prevent infection by and spread of MDR GNB with regard to utilization of newer as well as older agents, has not yet been determined. It is, therefore, imperative for clinicians to use carbapenems and other broad spectrum antibiotics judiciously in order to prolong the lifespan of these valuable drugs. Finally, studies need to focus on use of newer agents in complex patient populations with MDR GNB infection for better understanding of dosing, toxicity and clinical efficacy. Development of rapid diagnostics to identify causative microorganisms, resistance mechanisms, and antimicrobial susceptibility is warranted to help in the choice of appropriate antimicrobial therapy.

## Figures and Tables

**Table 1:** Percentage of susceptible, intermediate, and resistant isolates of selected Gram-negative bacilli when tested against 13 antimicrobial agents.

Isolates (n)	MIC_50_/_90_	Antibiotic * (MIC μg/mL)												
		AN	AZM	C/T	FEP	CAZ	CZA	CIP	COL	FOS	IMP	MEM	TZP	TOB
***E. coli*** **(46)**	MIC_50_	8	32	0.25	128	16	0.25	32	0.5	4	0.25	0.06	4	2
	MIC_90_	16	64	32	128	128	8	32	1	32	2	0.25	128	64
***K. pneumoniae*** **(39)**	MIC_50_	8	128	2	128	128	0.5	32	0.5	256	16	0.125	64	16
	MIC_90_	512	128	128	128	128	128	32	1	512	128	128	512	128
***P. aeruginosa*** **(48)**	MIC_50_	64	16	128	64	64	32	16	1	128	64	32	128	64
	MIC_90_	128	32	128	128	128	64	32	1	512	128	128	256	128

* MIC, minimum inhibitory concentration, AN—amikacin, AZM—aztreonam, C/T—ceftolozane/tazobactam, FEP—cefepime, CAZ—ceftazidime, CZA—ceftazidime/avibactam, CIP—ciprofloxacin, COL—colistin, FOS—fosfomysin, IMP—imipenem, MEM—meropenem, TZP—piperacillin/tazobactam, TOB—tobramycin.

**Table 2:** Comparative activity of 13 antimicrobial agents against selected Gram-negative bacilli.

Isolates (n)	SIR ^a^	Antibiotics ^b^ (%)												
		AN	AZM	C/T	FEP	CAZ	CZA	CIP	COL	FOS	IMP	MEM	TZP	TOB
***E. coli*** **(46)**	S	91.3	13.0	76.1	13.0	23.9	89.1	30.4	95.7	97.8	84.8	89.1	65.2	54.3
	I	4.3	6.5	6.5	-	13.0	-	-	-	-	4.3	-	13.0	6.5
	R	4.3	80.4	17.4	87.0	63.1	10.9	69.6	4.3	2.2	10.9	10.9	21.7	39.1
***K. pneumoniae*** **(39)**	S	64.1	5.1	51.3	7.7	5.1	64.1	43.6	92.3	7.7	56.4	64.1	35.9	30.8
	I	5.1	-	2.6	-	-	-	2.6	-	12.8	7.7	-	15.4	5.1
	R	30.8	94.9	46.1	92.3	94.9	35.9	53.8	7.7	79.5	35.9	35.9	48.7	64.1
***P. aeruginosa*** **(48)**	S	31.2	47.9	33.3	25.0	22.9	39.6	16.7	97.9	-	10.4	8.3	14.6	31.2
	I	2.1	27.1	-	8.3	4.1	-	8.3	-	-	8.3	4.2	29.2	2.1
	R	66.7	25.0	66.7	66.7	73.0	60.4	75.0	2.1	-	81.3	87.5	56.2	66.7

^a^ S, susceptible; I, intermediate; R, resistant; ^b^ Refers to Table 1; AN—amikacin, AZM—aztreonam, C/T—ceftolozane/tazobactam, FEP—cefepime, CAZ—ceftazidime, CZA—ceftazidime/avibactam, CIP—ciprofloxacin, CL—colistin, FOS—fosfomycin, IMP—imipenem, MEM—meropenem, TZP—piperacillin/tazobactam, TOB—tobramycin.
